# Performance Assessment of Out-of-Hospital Use of Pelvic Circumferential Compression Devices for Severely Injured Patients in Switzerland: A Nationwide Retrospective Cross-Sectional Study

**DOI:** 10.3390/jcm12175509

**Published:** 2023-08-24

**Authors:** Lionel Balet, François-Xavier Ageron, Mathieu Pasquier, Tobias Zingg, The Swiss Trauma Registry

**Affiliations:** 1 Faculty of Biology and Medicine, University of Lausanne, 1005 Lausanne, Switzerland; 2 Department of Emergency Medicine, Lausanne University Hospital and University of Lausanne, Rue du Bugnon 46, 1011 Lausanne, Switzerland; 3 Department of Visceral Surgery, Lausanne University Hospital and University of Lausanne, Rue du Bugnon 46, 1011 Lausanne, Switzerland

**Keywords:** PCCD, pelvic fracture, emergency, pre-hospital

## Abstract

Background: Patients with severe pelvic fractures carry a greater risk of severe bleeding, and pelvic compression devices (PCCD) are used to stabilize the pelvis on the pre-hospital scene. The aim of this study was to describe the use of PCCD in the pre-hospital setting on a nationwide scale (Switzerland) and determine the sensitivity, specificity and rates of over- and under-triage of the current application practices. The secondary objective was to identify pre-hospital factors associated with unstable pelvic fractures. Methods: Retrospective cross-sectional study using anonymized patient data (1 January 2015–31 December 2020) from the Swiss Trauma Registry (STR). Based on AIS scores, patients were assigned a unique principal diagnosis among three categories (unstable pelvic fracture—stable pelvic fracture—other) and assessed for use or not of PCCD. Secondarily, patient characteristics, initial pre-hospital vital signs, means of pre-hospital transport and trauma mechanism were also extracted from the database. Results: 2790 patients were included for analysis. A PCCD was used in 387 (13.9%) patients. In the PCCD group, 176 (45.5%) had an unstable pelvic fracture, 52 (13.4%) a stable pelvic fracture and 159 (41.1%) an injury unrelated to the pelvic region. In the group who did not receive a PCCD, 214 (8.9%) had an unstable pelvic fracture, 182 (7.6%) a stable pelvic fracture and 2007 (83.5%) an injury unrelated to the pelvic region. The nationwide sensitivity of PCCD application was 45.1% (95% CI 40.1–50.2), the specificity 91.2% (95% CI 90–92.3), with both over- and under-triage rates of 55%. The prevalence of unstable fractures in our population was 14% (390/2790). We identified female sex, younger age, lower systolic blood pressure, higher shock index, pedestrian hit and fall ≥3 m as possible risk factors for an unstable pelvic fracture. Conclusions: Our results demonstrate a nationwide both over- and under-triage rate of 55% for out-of-hospital PCCD application. Female gender, younger age, lower blood pressure, higher shock index, pedestrian hit and fall >3 m are possible risk factors for unstable pelvic fracture, but it remains unclear if those parameters are relevant clinically to perform pre-hospital triage.

## 1. Introduction

Unintentional injury is one of the leading global causes of death and disability worldwide [1]. In Switzerland, the situation is not very different with accidents and violence as the principle causes of death among the population 25 to 44 years old [2]. Severe head injury and exsanguination are the major pathophysiological mechanisms of death in trauma patients [3].

Patients with pelvic fractures, particularly complex pelvic ring injuries, carry a greater risk of severe bleeding with a high associated mortality rate [4,5]. To improve the outcome of these patients, bleeding control is the cornerstone of pelvic trauma management [6]. The main sources of bleeding are from bony fracture surfaces and the sacral venous plexus [7]. It is therefore widely recognized that the early mechanical stabilization of a pelvic fracture is a central part of pre-hospital management [8,9,10,11]. To achieve this goal and limit blood loss, the initial pelvic strap-belt, first described by Vermeulen et al. [12], and subsequent types of pelvic circumferential compression devices (PCCD) have supplanted other pelvic devices such as the Pneumatic Antishock Garment (PSAG) or external fixators, and are now widely used in the pre-hospital phase of patient management [6,10,13]. In addition to stabilizing the pelvic ring, these compressive devices aim at reducing the pelvic volume and increase the intra-pelvic pressure with the goal of producing a tamponade effect [14,15,16]. According to Tile [17], the ligamentous stability of the pelvic ring is provided by the symphysis pubis, the posterior sacroiliac complex and the pelvic floor. Tile reported a relative contribution of the anterior structures (anterior to acetabulum) to the total stiffness of the pelvis of 40% and 60% for the posterior structures (posterior to acetabulum). His classification into three main types of pelvic fracture types (A, B, C) is based on the stability of the posterior structures (sacroiliac complex and pelvic floor), with stability being defined as the ability of the pelvis to withstand physiologic force vectors without deformation. At one end of the spectrum are type A fractures with an intact and therefore stable pelvic ring and at the other end of the spectrum are type C fracture with a completely (rotationally and translationally) unstable pelvic ring. Type B fractures fall between these two extremes with only partial (translational) stability of the pelvis. From this biomechanical perspective, only type B and C pelvic fractures will potentially benefit from PCCDs, as opposed to stable fracture types A [17]. However, the identification of unstable pelvic fractures in the pre-hospital setting is challenging. PCCDs are often either applied on patients with stable pelvic fractures, or omitted in the presence of unstable fractures. Hermans et al. [18], Yong et al. [19] and Schweigkofler et al. [20] reported very poor sensitivities and specificities in the application of pelvic binders in the pre-hospital scene. A recent study conducted by Zingg et al. [21] on trauma patients admitted to the Emergency Department of a Swiss tertiary university hospital observed that only 14% of patients with a PCCD placed in the field had a final diagnosis of type B or C pelvic fracture while among those with type B and C fractures, 25% had not received a pre-hospital PCCD. Since reliably excluding a mechanically unstable pelvic fracture in the pre-hospital setting is virtually impossible, PCCD application is often based on unspecific symptoms such as pain in the pelvic region. Injuries such as proximal femur or acetabular fractures are therefore at risk of inappropriate treatment with a pelvic binder, since this may increase pain and worsen fracture dislocation. When a treatment tool such as a PCCD may either provide significant benefit or harm, depending on the right indication, its application practices should be critically monitored.

The main purpose of our study was to describe the use of PCCD in the pre-hospital setting on a nationwide scale (Switzerland) and determine the over- and under-triage rates of the current application practice. The secondary objective was to identify pre-hospital factors associated with unstable pelvic fractures.

## 2. Materials and Methods

### 2.1. Study Design and Setting

This is a retrospective cross-sectional study using anonymized patient data from the Swiss Trauma Registry (STR). In Switzerland, trauma care is a domain of highly specialized medicine, which implies that only hospitals with trauma-specific facilities, designated by the federal government, can provide 24/7 care. Since 2010, twelve hospitals cover the national need. The pre-hospital rescue system consists of ground vehicle- or helicopter-based patient transport by qualified paramedics, assisted by emergency physicians.

### 2.2. Study Population and Data Collection

Anonymous data were extracted from the STR database, which collects data of severely injured patients (Injury Severity Score ≥ 16 and/or AIS head ≥ 3) older than 16 years old which were admitted to one of the twelve dedicated Swiss trauma centers. The study period covered the time from 1 January 2015 to 31 December 2020. Mass casualties, inter-hospital transfers, patients aged <18 years, ISS < 16 and cases with missing information regarding diagnoses were excluded. Patient characteristics, initial pre-hospital vital signs, means of pre-hospital transport, trauma mechanism and final diagnoses were extracted from the database.

### 2.3. Outcome Measures

Based on AIS scores for each injury, patients were assigned a unique principal diagnosis among three categories: 1. “B/C” which encompasses pelvic fracture types B or C according to the Tile classification [17]; 2. “proximal” which encompasses either pelvic fractures type A or proximal femoral/hip injuries (regional injury with stable pelvic ring); and 3. “other” which encompasses all injuries outside the pelvic region. Tile type A pelvic ring fractures are defined as injuries which do not disrupt the bony or ligamentous pelvic ring but may avulse portions of the pelvis or fracture the iliac wing. In this type of injury, the pelvic ring per se remains mechanically stable. In type B fractures, there is a rotational, but no vertical, mechanical instability. In type C fractures, the posterior disruption of the pelvic ring is complete, resulting in rotational and vertical instability [17]. Due to the potential pelvic soft-tissue—including vascular—injuries that occur at the moment of kinetic energy transfer at impact, Type B and C fractures are therefore often accompanied by significant bleeding. The primary outcome of the present study was the presence of an unstable pelvic fracture (type B or C fractures) and whether or not the use of PCCD was appropriate; application of the device in type B or C fractures was considered as beneficial.

### 2.4. Statistical Analysis

All statistical analyses were conducted using Stata version 17.0 (College Station, TX, USA). We first compared the group with PCCD documentation available with the PCCD undocumented group to check whether the two groups present disparities that could constitute a selection bias in the complete cases analysis. This was not the case, and the complete cases analysis could be used as representative of the entire study population. We then determined the sensitivity and specificity of the out-of-hospital PCCD application in Switzerland and further by trauma center. We compared patients receiving a PCCD to patients not receiving a PCCD by age, sex, ISS, NACA score, GCS score, cervical spine immobilization, intubation, presence of physician on site, time from scene to shock room, systolic blood pressure, heart rate, shock index, oxygen saturation, respiratory rate, means of transport and trauma mechanism. The statistical significance of the differences was assessed using a two-sample *t*-test for continuous variables with normal distribution and a two-sample Wilcoxon rank-sum test for non-parametric distribution. The chi-square test was utilized for categorical variables. A *p*-value of <0.05 was considered as statistically significant.

In a second step, using the same variables, we compared patients with Tile B/C fractures to patients without unstable pelvic fractures (proximal) and patients having injuries unrelated to the pelvis (other). Then, we performed an ANOVA comparison with Bonferroni correction or chi-square test on useful pre-hospital parameters to identify factors which could discriminate between unstable and stable pelvic fractures.

## 3. Results

**Figure 1:**
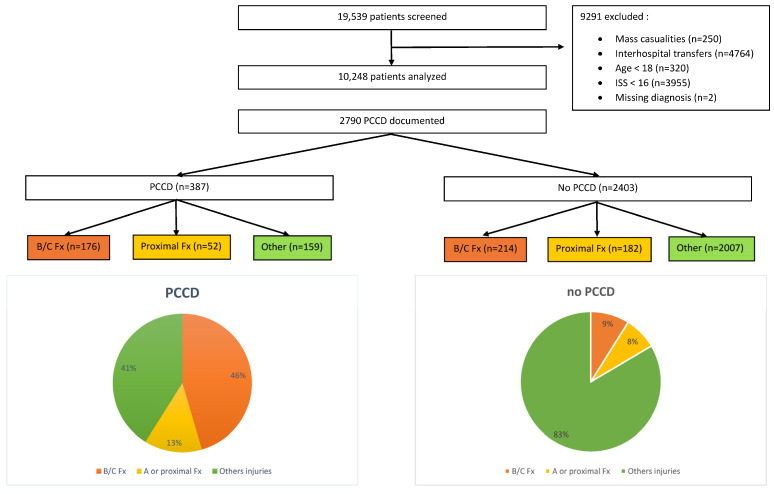
Inclusion flow chart and distribution of PCCD in documented cases. PCCD: Pelvic Circumferential Compression Device; B/C: Tile type B or C; proximal: Tile type A or proximal femoral/hip injuries; other: other injuries.

**Figure 2:**
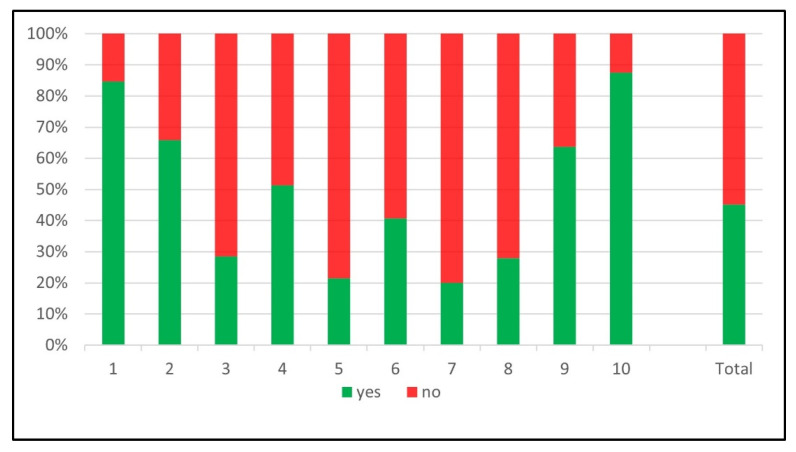
Percentage of application of PCCD in unstable pelvic fracture by trauma center.

**Figure 3:**
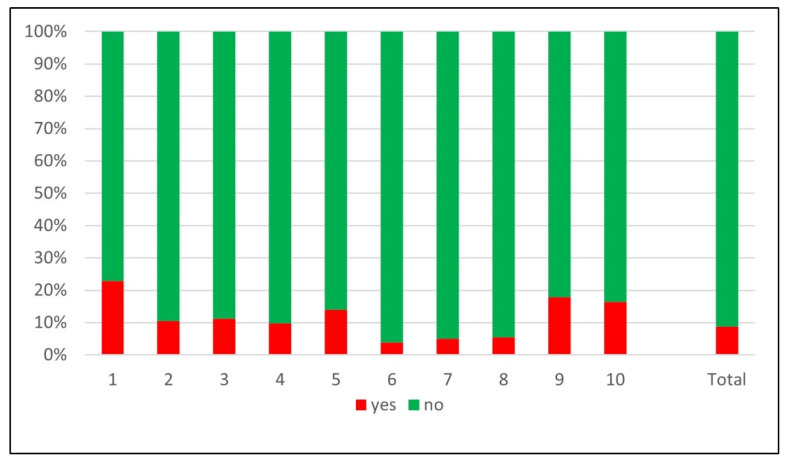
Percentage application of PCCD in stable pelvic fracture by trauma center.

**Figure 4:**
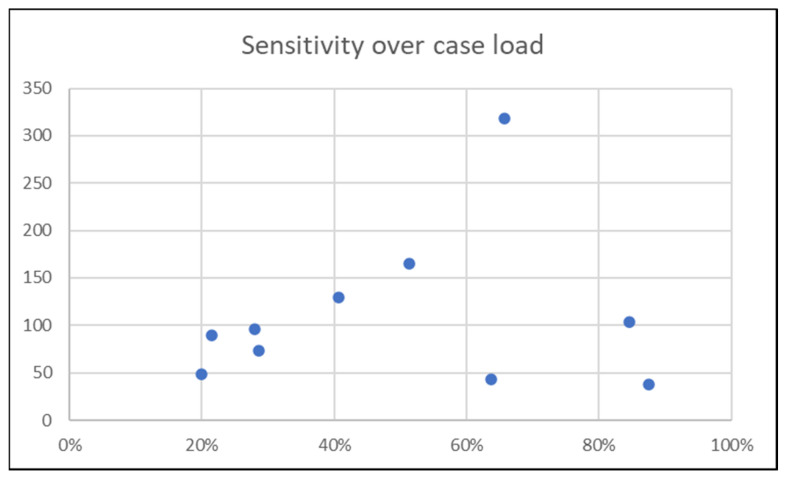
Sensitivity of PCCD application (*x*-axis) by trauma center and number of unstable pelvic fracture cases (*y*-axis).

Among the initial 19,539 patients screened in the STR, 9291 were excluded. Reliable information about PCCD application was documented in 2790 patients. Figure 1 shows the inclusion flowchart and distribution of PCCDs in documented cases. A PCCD was used in 387 (13.9%) patients. In the PCCD group, 176 (45.5%) had an unstable pelvic fracture, 52 (13.4%) a stable pelvic or proximal femur fracture and 159 (41.1%) an injury unrelated to the pelvic region. In the group who did not receive a PCCD, 214 (8.9%) had an unstable pelvic fracture, 182 (7.6%) a stable pelvic or proximal femur fracture and 2007 (83.5%) an injury unrelated to the pelvic region. The nationwide sensitivity of PCCD application was 45.1% (95% CI 40.1–50.2) and the specificity was 91.2% (95% CI 90–92.3). The prevalence of unstable fractures in our population was 14% (390/2790). The sensitivities and specificities of PCCD application by center are summarized in Figure 2 and Figure 3. The sensitivity ranges from 20.0% (4/20) to 87.5% (7/8) and the specificity from 77.1% (74/96) to 96.1% (671/698). Figure 4 shows the sensitivity of PCCD application related to the total number of unstable pelvic fracture cases (entire population, that is, PCCD-documented and PCCD-undocumented cases). The trauma center with the lowest sensitivity also has the lowest case load. A linear increasing trend between sensitivity and case load can be observed, except in three centers which show a sensitivity above average despite a small case load. The center with the highest sensitivity had the smallest case load. Two centers did not provide any data about PCCD application in the pre-hospital setting. The resulting global over- (211/387) and under-triage (214/390) rates of the PCCD application practice in the present cohort were identical at 55%.

The characteristics of the groups with and without PCCDs are summarized in Table 1. In the PCCD group, the patients were younger, had higher ISS and NACA scores, were more often intubated, received cervical spinal immobilization more often and a physician was more frequently present at the scene of the accident. Concerning initial vital signs, patients which received a PCCD had a lower systolic blood pressure, higher heart and respiratory rates and a higher shock index. We found no differences for sex, time from pre-hospital emergency intervention to arrival in the trauma room, oxygen saturation or GCS between the two groups. For the mean of pre-hospital transport, a difference was noted between the two groups, patients with PCCD being more often transported by helicopter and patients without PCCDs by ambulance without physician staffing. For all trauma mechanisms other than “falls from own height or less than 3 m” and “other”, patients received a PCCD significantly more often than not.

**Figure 5:**
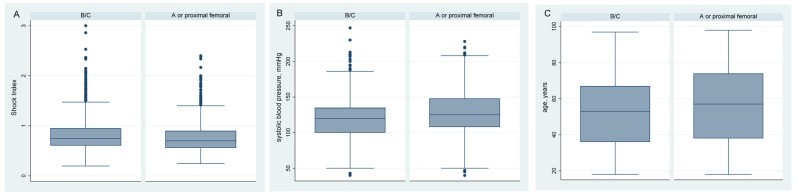
(**A**) Distribution of shock index (*p* < 0.001), (**B**) systolic blood pressure (*p* < 0.001) and (**C**) age (*p* < 0.001) in unstable and stable pelvic fracture groups. B/C: Tile type B or C; A or proximal: Tile type A or proximal femoral/hip injuries.

Table 2 summarizes the patient parameters, transport to hospital and trauma mechanism of the three groups by final diagnosis with regard to pelvic fracture. Patients with unstable pelvic fractures tend to be younger and male sex is less often represented in this group. In the group with unstable pelvic fractures, patients show a lower systolic blood pressure (118 mmHg vs. 126 mmHg) and a higher shock index (0.83 vs. 0.78). For trauma mechanism, pedestrians and falls > 3 m are more often represented in the group with unstable pelvic fractures. No difference between the unstable and the stable group was noted for NACA index, heart rate, respiratory rate, SaO_2_ and GCS score. Figure 5 illustrates the distribution of shock index, systolic blood pressure and age among the unstable and stable pelvic fracture groups.

## 4. Discussion

In the present study, the global nationwide sensitivity of PCCD application was 45%, which is comparable to the findings of a similar study from the US conducted by Bangura et al. [22] which found a 52% sensitivity. This is consistent with other studies from Agri et al. [23] and Schweigkofler et al. [20]. Based on the Germany Trauma Registry (DGU), Esmer et al. [24] conducted a study on over 33,000 patients to evaluate the value of the clinical pre-hospital assessment of pelvic injury type and severity. They found a sensitivity to detect severe pelvic injuries of 57%. However, other studies have shown sensitivities ranging from 21% to 75% [18,21,25] for their respective PCCD application practices. These disparate results are also reflected in the sub-group analysis by trauma center (20% to 87%) in our study. The interpretation of these results requires caution because different definitions and classifications for pelvic injuries were used among the prior studies. Furthermore, the radiological classification of pelvic fractures suffers from poor intra- and inter-observer agreement [26]. We can, however, highlight that the clinical diagnosis of unstable pelvic fracture is not reliable, as reported by Shlamovitz et al. [27] and van Leen et al. [28], in contradiction with the meta-analyses by Sauerland et al. [29] and Okada et al. [30]. As mentioned by Lustenberger et al. [31] in their study, it should be noted that “the vast majority of these studies were performed during the in-hospital resuscitation period” and not on scene, which precludes a relevant comparison with the purpose of our study. In their mini-review, Lee and Portner [10] also concluded that a number of studies on the accuracy of clinical diagnosis of pelvic fractures included different biases and, actually in the field, there is no reliable diagnostic test. In the present study, the sensitivity seems to be correlated with the total number of unstable fracture cases, except for three centers. It is difficult to estimate whether the differences in binder application performance among centers are a consequence of varying degrees of pre-hospital provider expertise or local application guidelines. This question was beyond the scope of the present paper, but may be addressed in future studies.

Concerning the characteristics between the populations with and without PCCD, we observed that patients with lower systolic blood pressure, higher heart and respiratory rates and higher shock index more often received a PCCD, although heart and respiratory rate do not appear to be different between patients with stable vs. unstable pelvic fractures according to our description of the two groups (Table 2). Increasing age was negatively correlated with PCCD use, and younger patients more often received a PCCD than the older, in accordance with the description of the unstable pelvic fracture group. Patients involved in road traffic accidents or sustaining falls from more than 3 m more often received a PCCD, although, according to the group description, only a fall from more than 3 m seems to be associated with an unstable pelvic fracture. Overall, it appears that the decision to apply a PCCD or not is influenced by the severity of the trauma (as evidenced by helicopter dispatch, presence of a physician, intubation and cervical spine immobilization all more prevalent in the PCCD group), regardless of whether the pelvis was involved or not. This is also reflected in the significant difference in final ISS (30 vs. 25) between the groups with and without a PCCD. Interestingly, Bangura et al. [22] and Lustenberger et al. [31], in their respective studies of pre-hospital PCCD use on pelvic fractures, found a negative correlation between ISS and PCCD application, with a lower ISS being a predictor of PCCD use. Bangura et al. [22] interpreted that, possibly, less severe trauma patients required less immediate care and allowed more time to examine and manage the pelvis. In these two studies focusing on pre-hospital PCCD use, patient intubation and time from emergency intervention to arrival in the trauma room were also in disagreement with our study. For Lustenberger et al. [31], intubation was positively correlated with PCCD omission, and in the study of Bangura et al. [22], an accident site in proximity to the hospital was a risk factor for omitting PCCD application, while, for us, no “time to trauma room” difference was found between the groups with or without a PCCD, and intubation was positively correlated with PCCD application. The etiology of trauma was also different between the study of Bangura et al. [22] and ours, with motor vehicle accidents accounting for more than 80% of pelvic fractures and falls for less than 5% in Bangura’s study, whereas in our study, motor vehicle accidents and falls each accounted for about 45% of unstable pelvic fractures.

To summarize, the description of patient characteristics, initial vital signs and trauma mechanism according to their final diagnosis highlights female gender, younger age, lower blood pressure, higher shock index, pedestrian hit and fall > 3 m as potentially relevant triage elements in pre-hospital care. Further studies are needed to determine the weight of each of these parameters, but we can notice that despite a significant *p*-value, the two groups are not so far different for the aforementioned parameters (Figure 5), which questions if those parameters are relevant clinical criteria in the field to correctly discriminate between an unstable pelvic fracture that could potentially benefit from pelvic compression devices and a stable pelvic fracture or proximal femoral fracture in which the pelvic belt could potentially be deleterious. Furthermore, with an average systolic blood pressure of 118 mmHg, average shock index under 0.9, average heart rate under 100/min and average respiratory rate under 20/min, some of the patients in the unstable pelvic fracture group are probably not in hemorrhagic shock but hemodynamically normal. If true, this would question the purpose of applying a PCCD to unstable pelvic fractures according to the idea that patients with complex pelvic fractures carry a greater risk of severe bleeding [4,5] that could be countered with the PCCD [8,9,10,11]. In their review of the pathophysiology and acute management of bleeding in pelvic fractures, Dyer and Vrahas [7] concluded that “there is broad consensus that bleeding is more likely to occur in unstable fractures. However, it remains difficult to predict which fractures will actually cause excessive bleeding”. This finding is consistent with the conclusion of Gustavo Parreira et al. [32] that there is no correlation between pelvic fracture type and mortality rate. A more recent study conducted by Ruatti et al. [5] on the relationship between fracture type and significant blood loss showed that there was no difference between Tile A and B fractures, but that there was a significant difference with Tile C fractures; this result was also found by Agri et al. [23]. A more accurate classification into fracture type and subtype therefore seems necessary to better predict which patients might benefit from PCCD in an effort to combat hemorrhagic shock. As noted above, however, it is highly unlikely that a sufficiently accurate diagnostic tool will emerge in the field to allow such a level of diagnosis. Thus, a liberal use of PCCD should therefore be proposed, as long as the benefits outweigh the risks associated with its use, this last point being conversely discussed in the literature [20,22,25]. In the present study, 13% of patients to whom a PCCD was applied were in the “Proximal” group, that is, a Tile type A fracture, or either a proximal femoral injury (neck or pertrochanteric), a hip dislocation or an acetabular fracture. In all these types of injury, a compression at the trochanteric level by application of the PCCD must be considered as harmful and contraindicated since it may cause further fracture displacement and increase pain, without any mechanical or physiological benefit. This is why more studies are needed to affirm the safety of PCCD application on stable pelvic fractures and to demonstrate its effectiveness in controlling bleeding in complex pelvic fractures.

Our study suffers from several biases inherent to its retrospective nature. Another main limitation was the small number of reported use or not of PCCD in patients suffering from unstable fractures among all the documented pelvic fracture types B or C (n = 1304). This represents 70% of the cases for which no PCCD data were available in the STR. Since the Swiss Trauma Registry only includes severely injured patients (defined by the presence of an ISS > 15 and/or an AIS head > 2), Tile type B and C fractures may be over-represented in our study population.

## 5. Conclusions

The primary objective of our study was to evaluate the performance of the out-of-hospital use of pelvic circumferential compression devices for severely injured patients in Switzerland. The study found a global sensitivity of 45.1% and a specificity of 91.2%, however with a great variability between individual centers. The global over- and under-triage rates of PCCD application were 55%, respectively. Descriptive analysis of patient characteristics according to diagnosis highlights female gender, younger age, lower blood pressure, higher shock index, pedestrian hit and fall > 3 m as possible risk factor for unstable pelvic fractures, but it remains unclear if those parameters are clinically relevant to optimize pre-hospital triage for PCCD application.

## Figures and Tables

**Table 1:** Patient parameters, transport to hospital and trauma mechanism according to Pelvic Circumferential Compression Device (PCCD). ISS = Injury Severity Score, NACA = National Advisory Committee for Aeronautics, GCS = Glasgow Coma Scale.

	Total (n = 2790)	PCCD (n = 387)	no PCCD (n = 2403)	*p*-Value
Patient characteristics				
Age, mean (SD)	57.5 (21.3)	47.0 (18.5)	59.2 (21.2)	<0.001
Male sex, n (%)	1996 (71.5)	283 (72.9)	1713 (71.3)	0.5
ISS, median (IQR)	24 (11)	27 (17)	22 (11)	<0.001
NACA score, mean (SD), (n = 1266)	4.3 (0.8)	4.7 (0.6)	4.3 (0.9)	<0.001
Cervical spine immobilization, n (%)	611 (46.9)	166 (77.6)	445 (40.8)	<0.001
Intubation, n (%)	671 (24.2)	121 (31.3)	550 (23.0)	<0.001
Physician on site of accident, n (%)	521 (65.0)	118 (85.0)	403 (60.8)	<0.001
Time to deschock min, mean (SD), (n = 2464)	44.8 (18.3)	45.7 (19.8)	44.6 (18.1)	0.35
Initial vital signs				
Systolic blood pressure mmHg, mean (SD), (n = 2436)	133.8 (32.3)	116.7 (29.3)	136.5 (31.9)	<0.001
Heart rate min-1, mean (SD), (n = 2501)	86.8 (21.7)	92.4 (24.8)	85.9 (21.0)	<0.001
Respiratory rate min-1, mean (SD), (n = 1347)	18.9 (8.3)	20.7 (9.4)	18.6 (8.1)	<0.001
Shock Index, mean (SD), (n = 2387)	0.7 (0.3)	0.8 (0.4)	0.7 (0.3)	<0.001
SaO2 %, median (IQR), (n = 2420)	95 (7)	95 (9)	95 (7)	0.58
GCS score, mean (SD), (n = 2694)	11.5 (4.3)	11.4 (4.5)	11.5 (4.3)	0.48
Means of transport	n = 2768	n = 387	n = 2381	
helicopter, n (%)	1001 (36.2)	226 (58.4)	775 (32.5)	<0.001
private, n (%)	44 (1.6)	0	44 (1.8)	-
ambulance with physician, n (%)	1049 (37.9)	133 (34.4)	916 (38.5)	0.12
ambulance without physician, n (%)	665 (24.0)	28 (7.2)	637 (26.7)	<0.001
other, n (%)	9 (0.3)	0	9 (0.4)	-
Trauma mechanism	n = 2732	n = 386	n = 2346	
pedestrian, n (%)	146 (5.3)	31 (8.0)	115 (4.9)	0.011
2 wheels, n (%)	633 (23.1)	116 (30.0)	517 (22.0)	0.001
4 wheels, n (%)	218 (8.0)	48 (12.4)	170 (7.2)	<0.001
fall from its height, n (%)	211 (7.7)	0	211 (9.0)	-
fall < 3 m, n (%)	680 (24.9)	23 (6.0)	657 (28.0)	<0.001
fall >= 3 m, n (%)	474 (17.3)	123 (31.9)	351 (15.0)	<0.001
other, n (%)	370 (13.5)	45 (11.7)	325 (13.8)	0.24

**Table 2:** Patient parameters, transport to hospital and trauma mechanism according to diagnosis. *p*-value applies for comparison between “B/C” and “proximal” groups. ISS = Injury Severity Score, NACA = National Advisory Committee for Aeronautics, GCS = Glasgow Coma Scale.

	Total (n = 10,248)	B/C (n = 1304)	Proximal (n = 889)	Other (n = 8055)	*p*-Value
Patient characteristics					
Age, mean (SD)	57.2 (21.2)	52.6 (20.1)	56.2 (21.4)	58.1 (21.3)	<0.001
Male sex, n (%)	7160 (69.9)	799 (61.3)	625 (70.3)	5736 (71.2)	<0.001
ISS, median (IQR)	24 (12)	29 (19)	22 (10)	22 (10)	
NACA score, mean (SD), (n = 1326)	4.3 (0.8)	4.5 (0.7)	4.3 (0.8)	4.3 (0.9)	0.27
Cervical spine immobilizatio, n (%)	622 (47.1)	115 (58.4)	62 (57.9)	445 (43.8)	
Intubation, n (%)	7630 (22.1)	261 (20.7)	179 (21.2)	1718 (22.4)	
Physician on site of accident, n (%)	539 (63.3)	97 (78.2)	51 (66.2)	391 (60.1)	
Time to deschock min, mean (SD), (n = 7397)	44.5 (19.0)	43.5 (18.7)	45.7 (19.9)	44.5 (19.0)	
Initial vital signs					
Systolic blood pressure mmHg, mean (SD), (n = 8298)	133.4 (31.6)	118.5 (29.1)	126.5 (32.6)	136.7 (31.1)	<0.001
Heart rate min-1, mean (SD), (n = 8594)	87.2 (21.6)	91.4 (22.9)	91.0 (22.2)	86.0 (21.1)	1.00
Respiratory rate min-1, mean (SD), (n = 4085)	18.9 (7.7)	19.8 (8.6)	19.7 (6.9)	18.6 (7.7)	1.00
Shock Index, mean (SD), (n = 8155)	0.7 (0.3)	0.8 (0.4)	0.8 (0.3)	0.7 (0.3)	<0.001
SaO2 %, median (IQR), (n = 7473)	96 (6)	95 (7)	95 (8)	96 (6)	0.58
GCS score, mean (SD), (n = 9059)	11.7 (4.2)	12.6 (3.7)	12.1 (4.0)	11.6 (4.3)	0.08
Means of transport	n = 10,088	n = 1292	n = 864	n = 7932	
helicopter, n (%)	3880 (38.5)	615 (47.6)	390 (45.1)	2875 (36.3)	
private, n (%)	377 (3.7)	12 (0.9)	9 (1.0)	356 (4.5)	
ambulance with physician, n (%)	3405 (33.8)	475 (36.8)	278 (32.2)	2652 (33.4)	
ambulance without physician, n (%)	2300 (22.8)	177 (13.7)	183 (21.2)	1940 (24.5)	
other, n (%)	126 (1.2)	13 (1.0)	4 (0.5)	109 (1.4)	
Trauma mechanism	n = 9824	n = 1284	n = 857	n = 7683	<0.001
pedestrian, n (%)	510 (5.2)	144 (11.2)	56 (6.5)	310 (4.0)	
2 wheels, n (%)	2159 (22.0)	272 (21.2)	213 (24.9)	1674 (21.8)	
4 wheels, n (%)	918 (9.3)	138 (10.8)	132 (15.4)	648 (8.4)	
fall from its height, n (%)	254 (2.6)	6 (0.5)	15 (1.8)	233 (3.0)	
fall < 3 m, n (%)	3038 (30.9)	159 (12.4)	142 (16.6)	2737 (35.6)	
fall >= 3 m, n (%)	1721 (17.5)	415 (32.3)	225 (26.3)	1081 (14.1)	
other, n (%)	1224 (12.5)	150 (11.7)	74 (8.6)	1000 (13.0)	

## Data Availability

The data are available from the corresponding author upon request.
